# Anti-Tumor Necrosis Factor-α Use in Pediatric Inflammatory Bowel Disease—Reports from a Romanian Center

**DOI:** 10.3390/ph18010084

**Published:** 2025-01-11

**Authors:** Roxana Matran, Andra-Mihaela Diaconu, Andreea Maria Iordache, Irina Dijmărescu, Alexandra Coroleucă, Daniela Păcurar, Cristina Becheanu

**Affiliations:** 1 Department of Paediatrics, “Carol Davila” University of Medicine and Pharmacy, 050474 Bucharest, Romania; elena.smadeanu@umfcd.ro (R.M.); irina.dijmarescu@umfcd.ro (I.D.); alexandra.coroleuca@umfcd.ro (A.C.); danapacurar@yahoo.com (D.P.); cbecheanu@yahoo.com (C.B.); 2 “Grigore Alexandrescu” Emergency Hospital for Children, 011743 Bucharest, Romania

**Keywords:** inflammatory bowel disease, anti-TNF-α, antidrug antibodies, acute-infusion reactions, adverse events

## Abstract

**Background/Objectives:** The introduction of anti-tumor necrosis factor-α (anti-TNF-α) agents, particularly infliximab (IFX) and adalimumab (ADA), has significantly expanded the therapeutic arsenal for inflammatory bowel disease (IBD). While these biologics have demonstrated substantial efficacy, they are associated with a spectrum of potential adverse events (AEs). This study aims to evaluate and document these AEs to facilitate optimal patient selection and monitoring strategies of patients undergoing these therapies. **Methods**: This retrospective, single-center study examined pediatric IBD patients receiving anti-TNF-α therapy at the “Grigore Alexandrescu” Emergency Hospital for Children in Bucharest, Romania, from January 2015 to October 2024. AEs were categorized into non-infectious complications (acute infusion reactions, anti-drug antibody formation), dermatological effects (erythema nodosum, vasculitis), neurological effects (Guillain–Barré syndrome), and infections. AEs were analyzed in relation to the specific anti-TNF-α agent administered and comprehensively characterized. **Results**: Of 40 patients enrolled, 22 (55%) had Crohn’s disease (CD). The median (IQR) age at diagnosis was 14.8 years [10.8–15.9]. IFX was used in 34 (85%) patients while 6 (15%) patients received either ADA or IFX/ADA sequential therapy. Twenty-seven AEs were documented in 19 (47.5%) patients, the most prevalent being antidrug antibody formation (44.4%), infections (22.2%), and acute infusion reactions (22.2%). All ADA-exposed patients experienced at least one AE, compared to 41.2% (n = 14) patients treated with IFX, *p* = 0.01. **Conclusions**: AEs were observed in approximately half of the study cohort, with anti-drug antibody formation emerging as the most frequent complication. ADA therapy was associated with a significantly higher rate of AEs compared to IFX. These findings underscore the critical importance of vigilant monitoring for patients undergoing anti-TNF-α therapy in pediatric IBD management.

## 1. Introduction

Inflammatory bowel disease (IBD), primarily comprising Crohn’s disease (CD) and ulcerative colitis (UC) [1], is characterized by a chronic, relapsing-remitting course [2]. The etiology of IBD remains elusive, but it is hypothesized to result from immune dysregulation and aberrant host-microbiome interactions in genetically susceptible individuals, triggered by specific environmental factors [3].

Over the past three decades, pediatric IBD incidence and prevalence have significantly increased, particularly in children under 5 years old, with a notable decrease in the age at diagnosis [4]. Approximately 25% of IBD cases manifest during childhood, with very-early-onset IBD (VEO-IBD) accounting for 20% of pediatric IBD diagnoses [5]. A systematic review by Ng et al. revealed that CD and UC prevalence is highest in developed regions such as North America and Western Europe, with emerging trends in developing countries [6]. Kuenzig et al. reported the highest incidence rates in Northern Europe, Canada, and New Zealand, contrasting with lower rates in Southern Europe, Africa, Asia, and South America. Most studies indicate a 2–3:1 ratio favoring CD over UC, particularly in Quebec (Canada) and New Zealand [7].

The therapeutic resources for IBD have expanded significantly over the past few decades. As Burger et al. note, treatment options were limited to medication like aminosalicylates (5-ASA), corticosteroids, and other immunosuppressive drugs like thiopurines (azathioprine and 6-mercaptopurine) and methotrexate [8]. Beginning more than 20 years ago, advances in understanding IBD pathogenesis and progress in the field of immunology have led to an expansion of treatment options [9]. This progress shifted treatment strategies towards a “treat-to-target” therapy, a concept that aims for both clinical and endoscopic remission [10].

Tumor necrosis factor alfa (TNFα) plays an essential role in the pathogenesis of IBD. It is a pro-inflammatory cytokine that is found in high concentrations within the lamina propria of the small intestine and colon of IBD patients [11]. Its role in IBD-related inflammatory processes emphasizes its potential as a key target for therapeutic interventions [12].

Monoclonal antibodies targeting TNFα in both circulating and tissue-bound forms include IFX, ADA, golimumab, certolizumab pegol, and etanercept [13]. However, only IFX and ADA have received Food and Drug Administration (FDA) and European Medicines Agency (EMA) approval for pediatric use [11].

IFX is a chimeric monoclonal antibody (25% murine-derived) that was approved by the FDA for pediatric CD in 2006 [14] and UC in 2011 [15]. It binds both soluble and transmembrane TNFα, blocking its activity [16]. The standard dose is 5 mg/kg via intravenous infusion [17]. The standard treatment regimen consists of an induction phase at weeks 0, 2, and 6, followed by maintenance every 8 weeks, although dose intensification or shorter intervals may be required in younger children or to maintain therapeutic drug levels [18,19].

ADA, a fully humanized recombinant monoclonal IgG1 antibody, received FDA approval for pediatric CD in 2012 [20] and recently for UC [21]. European Crohn’s and Colitis Organization (ECCO)/ESPGHAN guidelines recommend subcutaneous administration: 2.4 mg/kg initially, 1.2 mg/kg at week 2, and 0.6 mg/kg every other week for maintenance [17].

Pediatric IBD treatment strategies typically follow expert recommendations from pediatric gastroenterology organizations. The usual approach involves incrementally intensifying treatment based on response to previous medications. However, current trends favor risk stratification, leading to the earlier introduction of biologics (a “top-down” approach) in high-risk patients [22]. Treatment selection also considers local guidelines and therapy availability.

Anti-TNF agents have dramatically improved outcomes in pediatric IBD and other autoimmune disorders. However, their use is associated with potential adverse events [23]. Consequently, careful patient selection [17] and ongoing monitoring are essential for optimal therapeutic management [24].

This study addresses a critical gap in the literature by focusing on the adverse events (AEs) associated with anti-TNF therapy in pediatric IBD patients within our regional context. Despite the increasing use of these biological agents in pediatric IBD management, there is a notable paucity of data from our country. This research aims to comprehensively assess and document the AEs encountered in our clinical practice, thereby enhancing our ability to anticipate, recognize, and effectively manage these potential complications. The findings of this study will contribute valuable regional-specific data to the existing body of knowledge, potentially informing more tailored approaches to pediatric IBD management in similar healthcare settings.

## 2. Results

A total of 40 patients were enrolled, of whom 22 (55%) were diagnosed with CD. The median (IQR) age at diagnosis was 14.8 years [10.8–15.9] with four (10%) patients being classified as VEO-IBD. The median [IQR] age at enrollment was 17 years [14.2–17.9]. Regarding our center, we administered IFX for the first time in 2014 to a patient diagnosed with UC at the age of 5 years and 8 months, who began treatment with a biosimilar IFX molecule in Germany. Concerning the first administration of ADA, we used it in 2015 for a 16-year-old patient diagnosed with CD. Characteristics of the patients are described in Table 1.

**Figure 1:**
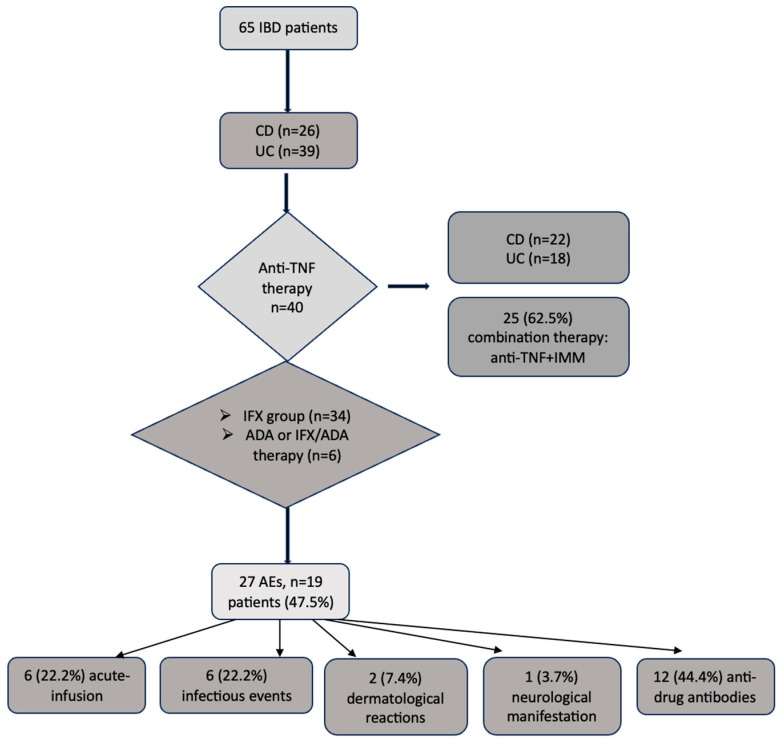
Flow-graph of the main data analysis. ADA, adalimumab; AEs, adverse events, CD, Crohn’s disease; IBD, inflammatory bowel disease; IFX, infliximab; IMM, immunomodulator; UC, ulcerative colitis; TNF, tumor necrosis alfa.

The schematic analysis of the data is presented in Figure 1.

During the observation period, the distribution of anti-TNF-α therapies among the study cohort was significantly skewed (*p* < 0.001). A predominant majority of patients (n = 34, 85%) received only IFX, while a smaller subset (n = 6, 15%) underwent either ADA or sequential IFX/ADA treatment. This distribution reflects therapeutic modifications implemented in response to AEs. Specifically, two patients transitioned from ADA to IFX, and one patient switched from IFX to ADA due to AE-related concerns. These therapeutic adjustments underscore the dynamic nature of IBD management and the necessity for individualized treatment approaches in the face of treatment-related complications.

Time from diagnosis to biologics initiation had a median (IQR) of 6.5 months [2.0–16.5] for the entire group, with 5.5 months [2.0–13.75] for CD and 7.5 months [1.25–15.75] for UC, *p* = 0.989. Younger patients tend to experience a longer delay in initiating biologic therapy compared to those diagnosed at an older age, (*p* < 0.001, R^2^ = 0.381). The median (IQR) IFX dose was 5.0 mg/kg/administration [5.0–6.75] for CD, while for UC it was slightly higher at 6.5 mg/kg/administration [5.0–10.0], with no statistical difference, *p* = 0.182. As for ADA administration, standard and optimized regimes were administered in equal proportions.

Twenty-five patients (62.5%) received combination therapy, including twelve (48%) patients with CD (*p* = 0.747), highlighting the frequent use of this treatment strategy in our cohort, irrespective of the disease type.

Out of the 25 patients under combination therapy, 12 (48%) patients presented with AEs, of which 8 (66.7%) patients received only IFX and 4 (33.3%) patients received either ADA or sequential IFX/ADA therapy, *p* = 0.248.

We draw attention to a noteworthy case involving a patient with ulcerative colitis (UC) who experienced two distinct AEs under different therapeutic regimens. The first AE, specifically the formation of anti-drug antibodies, occurred in the absence of combination therapy. Subsequently, the patient developed a second AE, manifesting as hypersensitivity vasculitis (HV), while undergoing combination therapy.

Twenty-seven AEs were documented in 19 (47.5%) patients. Out of the 27 AEs, 20 (74.1%) were as a consequence of IFX administration and 7 (25.9%) were in association with ADA or sequential IFX/ADA therapy, *p* = 0.01.

The 27 AEs included 6 (22.2%) acute infusion-related events, 5 of which were classified as severe; 6 (22.2%) infectious episodes were reported in five patients, including two varicella-zoster virus reactivations, one patient who developed recurrent *Clostridioides difficile* (*C. difficile*) infection in association with perianal condilomatosis with *Human papilloma virus* (*HPV*), one patient who developed severe measles complicated with pneumonia and respiratory failure, and one case of bronchopulmonary tuberculosis (TB). In addition, two (7.4%) dermatological reactions like *erythema nodosum* (EN) and HV were observed, and one (3.7%) neurological manifestation (Guillain–Barré Syndrome, GBS) was noted in an adolescent. Anti-drug antibody formation was the most prevalent AE, occurring in 12 (44.4%) patients. Detailed descriptive statistics for all AEs are presented in Table 2. Notably, all 6 patients that received ADA experienced at least one AE, compared to 14 patients (41.2%) exposed to IFX (n = 34), *p* = 0.01.

No deaths or malignancies were recorded at the time of completion of this study.

No statistical differences were observed between IFX and ADA therapy regarding the occurrence of specific AEs. The results are summarized (Table 3).

As for the ADA subgroup of patients, there were no significant differences between the standard vs. the optimized treatment strategies in terms of AE occurrence (Table 4).

Anti-drug antibodies were detected in 12 patients, with 66.7% (n = 8) receiving combination therapy and 33.3% (n = 4) on anti-TNF monotherapy at the time of detection (*p* = 0.221). The incidence of antibody formation was comparable between IFX and ADA groups under combination therapy, with 66.7% of patients developing antibodies in both groups (6/9 for IFX, 2/3 for ADA; *p* = 1). These findings suggest no significant association between therapeutic regimen and antibody formation, though the small sample size warrants cautious interpretation.

Although patients who developed anti-drug antibodies received higher median (IQR) IFX doses compared to those who did not (7.75 mg/kg/administration [5.75–10.0] vs. 5.0 mg/kg/administration [5.0–7.5], respectively), this difference did not reach statistical significance (*p* = 0.09). The median (IQR) time to antibody formation did not significantly differ between patients on combination therapy and those on monotherapy. For patients on combination therapy, the median time was 13.0 months [10.25–23.75], while for patients on monotherapy with anti-TNF molecules it was 13.5 months [9–36.25] (*p* = 0.932).

Infections were observed as an AE in five patients during the study period. Of these, four patients (80%) were receiving combination therapy, while one patient (20%) was on IFX monotherapy. This difference in infection rates between combination therapy and monotherapy approached but did not reach, statistical significance (*p* = 0.094). When analyzing infections by treatment type, both ADA-treated patients (100%) who developed infections were receiving combination therapy at the time of infection. In the IFX subgroup, two out of three patients (66.7%) who developed infections were under combination therapy, while one (33.3%) was on monotherapy. The difference in infection rates between ADA and IFX combination therapy groups was not statistically significant (*p* = 1). IFX dosing did not significantly impact infection rates. The median (IQR) dose of IFX was 5.0 mg/kg/administration [5.0–6.25] for the infection-positive group, compared to 5.0 mg/kg/administration [5.0–8.5] for those without any infectious events (*p* = 0.554).

Acute infusion reactions were observed in six patients, all of whom were receiving IFX. Among these patients, two (33.3%) were under combination therapy, while four (66.7%) were receiving IFX monotherapy, *p* = 0.386. The median (IQR) IFX dose for patients who developed acute reactions was 5.0 mg/kg/administration [5.0–6.5], compared to 5.0 mg/kg/administration [5.0–9.5] for those who did not experience acute reactions, *p* = 0.526.

Both of the patients with dermatological AEs were under combination therapy, one with each molecule. The GBS occurred in an ADA-standard monotherapy patient. The small number of cases precludes further statistical analysis.

## 3. Discussion

This study aims to elucidate the spectrum of AE associated with anti-TNF therapy in pediatric IBD. Our findings reveal a statistically significant disparity in the utilization of IFX and ADA between CD and UC patients. While this suggests an association between anti-TNF agent selection and IBD subtype, it is crucial to note that this observation is likely confounded by regulatory constraints, as ADA is exclusively approved for CD treatment in our country.

The findings of our investigation indicate that the administration of anti-anti-TNF agents is associated with a diverse array of AEs. This observation underscores the complex safety profile of these biologic therapies in the context of IBD management.

The most encountered reactions reported in the literature include (but are not limited to) infusion reactions, viral, bacterial, or fungal infections (including reactivation of some viruses/mycobacteria), but also neurological events, auto-immune disorders, dermatological manifestations, and malignancies. Recent studies have documented AE rates ranging from 10.7% to 67% in patients treated with anti-TNF agents, leading to treatment discontinuation in some cases [25,26]. Our study’s higher prevalence of AEs may be attributed to the inclusion of anti-drug antibody formation as an AE.

Global pharmacovigilance data from the WHO VigiAccess database revealed 1,403,273 AEs related to anti-TNF monoclonal antibodies, with adalimumab (ADA) accounting for 840,417 reports [27]. While our data shows that more AEs were associated with IFX administration compared to ADA or sequential IFX/ADA therapy, it is crucial to interpret these results in the context of the patient distribution between the two groups. Analysis suggests that, despite the larger absolute number of AEs in the IFX group, the smaller ADA group actually had a higher proportion of patients experiencing AEs, highlighting the importance of considering group sizes when interpreting data. This universal occurrence of AEs, while not statistically robust due to the constrained sample, warrants careful consideration and may suggest a potential trend worthy of further investigation in larger, more statistically powered studies.

Studies on pediatric patients with IBD and juvenile idiopathic arthritis have identified infusion-related reactions as significant AEs associated with anti-TNF therapy, particularly IFX. Lichtenstein et al. define these events as immediate-type reactions occurring during the drug infusion or shortly after their administration (1–2 h). The results of their systematic review indicate that IFX was associated with higher rates of infusion-related events compared to other anti-TNF drugs as they occurred in 5–23% of IBD patients [28].

Pastore et al. reported anaphylactoid reactions as the most frequent serious AE, typically occurring after a median drug exposure of 1.5 months [29]. Dan-Nielsen et al. observed a spectrum of infusion-related reactions in IFX-treated pediatric ulcerative colitis patients, ranging from severe (7%) to moderate (3%) and minor events (22%) [30]. Kolho et al. noted that while acute infusion reactions often occur early in treatment, younger children may experience them over an extended period, irrespective of concomitant therapies [31].

Similar to their findings, in our population all six patients with acute infusion-related events were under IFX treatment, irrespective of concomitant azathioprine use. One patient exhibited a minor reaction manageable through adjusted infusion rates, while five others developed moderate to severe reactions (e.g., angioedema, bronchospasm, tachycardia, vomiting, and urticaria) necessitating treatment discontinuation. One CD patient successfully transitioned to ADA. Notably, all patients received standard pre-medication with antihistamines and corticosteroids prior to IFX infusions.

Secondary loss of response, a significant concern in pediatric IBD treatment with biologics, can lead to premature therapy discontinuation. This phenomenon is often associated with increased immunogenicity of anti-anti-TNF agents. Corica et al. report that this immunogenicity can result in anti-drug antibody formation, potentially neutralizing the biologic agent or accelerating its clearance [32]. The formation of anti-drug antibodies in response to anti-TNF therapy was first reported by Elliott et al. 30 years ago in rheumatoid arthritis patients, with approximately half developing antibodies to the murine portion of the drug [33]. Subsequent studies have shown variable incidence rates: Vermeire et al.’s systematic review reported 0–65.3% for IFX and 0.3–38% for ADA [34]. A meta-analysis by Thomas et al. focusing on IBD patients found a cumulative incidence of 15.8% (95% CI 9.6–24.7) for anti-TNF antibodies, predominantly studying IFX [35]. Recently, Winter et al. detected anti-TNF antibodies in 17% of pediatric IBD patients while investigating potential biomarkers for treatment efficacy [36].

In our study cohort, we speculate that the main reason for the higher incidence of anti-drug antibodies in both IFX and ADA-treated patients was the reactive measurement strategy employed in cases of loss of response. To counteract antibody presence, dose escalation and shortened administration intervals were primarily utilized. In two cases of CD, therapy was switched from ADA to IFX as a subsequent treatment option.

The current literature suggests the addition of IMM to IFX therapy to mitigate antibody formation [35,37]. However, evidence regarding the efficacy of combination therapy versus monotherapy in pediatric IBD remains conflicting [38,39,40]. A systematic review by Corica et al. found no significant differences between these approaches in terms of anti-IFX antibody formation [32]. The efficacy of IMM addition for reducing antidrug antibody formation in CD patients treated with ADA remains a subject of ongoing debate [41]. In our study, the formation rates of antibodies were comparable regardless of the concomitant use of IMM.

GBS is an acute, autoimmune, and demyelinating polyradiculoneuropathy. Wachira et al. describe the clinical picture of this disease as ascending progressive weakness, associated with diminished or absent reflexes, culminating with a state of acute flaccid paralysis [42]. While GBS has been reported in patients receiving biologic treatments for IBD, these cases are rare and have been primarily documented in adults [43,44,45].

To our knowledge, we report the first pediatric case of GBS associated with ADA treatment for CD. A 17-year-old female receiving first-line ADA therapy underwent terminal ileum resection due to treatment failure. Postoperatively, she rapidly developed dysphagia and lower limb weakness, requiring mechanical ventilation. Electromyography confirmed acute inflammatory polyneuropathy with segmental demyelination, consistent with GBS. Treatment included plasmapheresis and high-dose intravenous immunoglobulins. After three weeks in intensive care and subsequent neurological care, the patient recovered slowly. ADA and other biologic agents were permanently discontinued.

Establishing a causal relationship between GBS and ADA is complex due to potential confounding factors. While post-surgical GBS is rare [46], our case suggests a possible link between ADA administration and GBS development. This hypothesis is supported by the observed demyelinating subtype, which contrasts with the more common axonal subtypes typically seen in post-surgical GBS [47]. Consequently, we propose that the GBS in our patient is more likely ADA-related rather than a post-surgical complication.

Multiple studies demonstrate a strong association between anti-TNF therapy and increased infection rates in IBD patients [29,48]. Day et al. suggest that this increased infectious burden is primarily due to immunosuppressive treatments rather than the underlying disease, except in VEO-IBD [49]. Manufacturers of IFX and ADA have highlighted elevated infection risks, particularly when combined with other immunosuppressants, leading to an FDA “Black Box Warning” in 2008 [50,51,52]. A systematic review by Dulai et al. reported an absolute rate of serious infections of 352 per 10,000 person-years in pediatric IBD patients receiving anti-TNF therapy, comparable to rates observed with IMM alone [53]. The REACH and IMAgINE studies reported overall infection rates of 54.5% and 67%, respectively, in pediatric CD patients treated with biologics [54,55]. Toussi et al.’s systematic review of pediatric IBD patients exposed to ADA or IFX found mild upper respiratory tract infections were most common (3–77% incidence), while severe infections were less frequent (0–10%). The wide range of reported infection rates may be attributed to inconsistent case definitions, varying study designs, treatment regimens, comorbidities, and reporting methods [56].

Ardura et al. demonstrate that the risk of infectious complications associated with anti-TNF therapy is time-dependent, with peak vulnerability occurring within the first 3–6 months of treatment initiation. The overall risk profile varies according to the specific anti-TNF agent and duration of exposure [24]. This finding is similar to the one observed in the present research.

In our study, the overall incidence of infections is 12.5% and includes moderate to severe infections of various etiology. These findings are consistent with previous reports. For instance, Szymanska et al. reported a similar infection rate of 12.2% in Polish CD pediatric patients. They primarily note respiratory and digestive infections, as well as one case of oral candidiasis [57]. Our results, although indicating a comparable rate of infections, include a broader range of observed infections of different types and severity.

Cullen et al.’s review of rheumatological studies found that viral infections constitute 30% of all infections and 11% of serious infections in anti-TNF users, with varicella-zoster virus (VZV) being a particular concern for IBD patients [58]. Schreiner et al. reported VZV reactivation in pediatric IBD patients, noting incidence variations based on gender and IBD subtype [59]. Veres et al. observed a 3.7% rate of herpes zoster in adolescents with IBD using IFX [60], comparable to our study’s 4.8% incidence, suggesting consistency with the existing literature on herpes zoster risk in pediatric IBD patients receiving anti-TNF therapy.

We experienced endogenous reactivation of VVZ in two previously unvaccinated patients, one UC patient treated with IFX monotherapy with an optimized dose (8 mg/kg), and one CD patient treated with combination therapy (ADA, optimized regimen in combination with azathioprine for almost 3 years). An important contributing factor was likely the lack of VVZ vaccination in children in our country’s National Immunization Program, which means we witness high incidence rates of infection, especially in children under 9 years of age [61]. Biologic agents and azathioprine were temporarily discontinued and resumed after specific antiviral treatment for both patients.

Findings from Dorhoi et al. reveal that TNFα has a crucial role in the immune response against *M. tuberculosis*. TNFα is essential for granuloma formation which represents aggregations of specialized macrophages and lymphocytes that play a vital role in restricting the dissemination of *M. tuberculosis* from different sites [62].

Anti-TNF therapy in adults with rheumatological diseases and IBD is associated with an increased risk of tuberculosis (TB) reactivation. The North American Society of Pediatric Gastroenterology, Hepatology and Nutrition (NASPGHAN) reported a 4 to 5-fold higher risk of TB reactivation in patients receiving anti-TNF therapy compared to those not receiving these medications. Anti-TNF agents independently contribute to this elevated risk, and combination therapy with other IMM further increases the likelihood of TB reactivation [24].

According to Romania’s National Strategy on Tuberculosis Control, Romania holds the highest burden of TB among European Union (EU) members, including pediatric and multidrug-resistant (MDR) TB. The report states that Romania accounts for 23.5% of the total TB cases reported in the EU, with a pediatric incidence of 12.8% in 2019 [63].

Limited data exist on TB risk in IBD patients receiving anti-TNF therapy. Cruz et al. reported the first two cases of disseminated TB associated with IFX use in pediatric IBD a decade ago [64]. Recently, two additional cases were described: a 13-year-old CD patient developing pulmonary TB after sequential immunosuppressive therapy [65], and a 12-year-old CD patient diagnosed with disseminated TB and Poncet’s disease following similar treatment [66]. While pediatric patients under anti-TNF treatment, particularly those with CD and juvenile idiopathic arthritis [67], are considered at risk for severe TB, a systematic review and meta-analysis by Kedia et al. on adult IBD patients suggests that TB risk correlates more closely with local TB prevalence than with specific treatment regimens [68].

We report the case of a 13-year-old female with refractory CD who developed severe bronchopulmonary TB 30 months after initiating ADA treatment, despite negative interferon-gamma release assay (IGRA) screenings. The patient received combination therapy of optimized ADA (dose augmentation and weekly administration) with azathioprine. TB occurrence, typically prevalent in the first three months of anti-TNF therapy, was most probably precipitated in our case by the recent intensification of her therapeutic regimen. Determining whether this was latent TB reactivation or new infection is challenging, considering the patient’s immunosuppressive regimen, repeated hospitalizations, and low socioeconomic background. The high TB prevalence in the country is noteworthy. ADA was discontinued and tuberculostatic treatment was administered. Biologics were resumed after two years, with IFX initiated due to chronic active CD evolution with perianal involvement despite ADA and azathioprine combination therapy.

HPV types 6 and 11 are associated with *condylomata acuminata*, primarily transmitted through sexual contact [69]. While the increased risk of HPV-associated cancer in IBD patients is well-documented, research on low-risk HPV infections in this population is limited [70]. Case reports suggest a potential link between anti-TNF therapy and genital HPV lesion development or exacerbation [71,72]. However, some studies indicate comparable anogenital wart risk among IBD patients regardless of treatment regimen [73,74].

*C. difficile* infection is a common complication in pediatric IBD patients [75]. Combination therapy with anti-TNF agents and other immunosuppressants may increase *C. difficile* infection risk [76]. IBD itself is an independent risk factor for *C. difficile* infection, particularly with colonic involvement. Corticosteroid use may increase *C. difficile* infection risk more than biologics or other IMM [77]. The presented case of severe, recurrent *C. difficile* infection combined with perianal condylomatosis in an unvaccinated adolescent, necessitated cessation of anti-TNF and IMM treatment, and fecal microbiota transplantation.

We report a case of a 17-year-old female with UC under combination therapy (IFX and azathioprine) who developed severe measles complicated by bronchopneumonia and acute respiratory failure 5 months post-IFX initiation. The patient was unvaccinated against measles-mumps-rubella (MMR), exacerbating her iatrogenic immunologic deficit. Severe acute colitis necessitated urgent immunosuppression, postponing vaccination and increasing infection risk. Treatment involved discontinuing IFX and azathioprine, administering immunoglobulins, and subsequently reintroducing IFX monotherapy due to persistent leukopenia. Following an acute infusion reaction, IFX was replaced with ADA. This case, potentially the first reported in pediatric IBD, underscores the risks of inadequate immunization in immunosuppressed patients, as corroborated by a similar adult case demonstrating compromised vaccine-induced immunity under immunosuppressive therapy [78].

The current literature presents conflicting evidence regarding infection risk in IBD patients receiving monotherapy versus combination therapy. While some studies suggest that combining immunosuppressive agents significantly increases infection risk [79], meta-analyses of randomized controlled trials indicate that combination therapy does not necessarily elevate overall adverse event rates, including infections [80,81,82]. The SONIC study found no significant increase in severe infection risk with combination therapy of IFX and azathioprine in CD [83]. Conversely, the Crohn Therapy, Resource, Evaluation, and Assessment Tool registry identified disease activity as the primary factor associated with serious infection risk [84]. Our observations suggest a higher percentage of infections with combination therapy, though lacking statistical significance due to the small sample size and thus limiting definitive conclusions.

HV affects small blood vessels and is characterized by its histopathological hallmark, namely leukocytoclastic vasculitis [85]. Anti-TNF-associated vasculitis pathogenesis may involve antibody formation against anti-TNF molecules or cytokine ratio disturbances [86]. Diagnosis is challenging in IBD cases, as vasculitis can be an extraintestinal manifestation. Symptom onset timing and disease activity assessment aid in determining etiology [87]. Giorgio et al. describe the case of anti-TNF-associated vasculitis which persisted through multiple treatments before responding to cyclosporin [88]. Early reports indicate a 36.4% incidence of anti-TNF vasculitis in UC patients using IFX, typically developing within 6–38 weeks of treatment initiation, despite standard administration protocols [89].

Our patient developed hypersensitivity vasculitis (HV) nearly 10 years after initiating IFX therapy, while in histological remission and receiving a standard IFX regimen. The condition manifested as palpable, nonpruritic purpura confined to the lower limbs, with normal immunological work-up. Notably, the patient had developed anti-IFX antibodies 4 years prior to the dermatological adverse event. The vasculitis persisted under continued IFX therapy due to family preference. While this timeline exceeds typical pediatric cases, similar delayed onset has been reported in adults [85]. The occurrence of HV during a period of ulcerative colitis inactivity strongly suggests a causal relationship with anti-TNF treatment.

EN is described as a septal panniculitis of the subcutaneous fat tissue resulting from a delayed hypersensitivity reaction [90]. While EN is a recognized extraintestinal manifestation of IBD [91] anti-TNF agents have also been implicated as potential triggers in adult studies [92,93]. Shivaji et al. suggest that the temporal relationship between biologic therapy initiation and EN onset is crucial in distinguishing between disease-related and treatment-induced EN [94]. Intriguingly, Zippi et al. note that anti-TNF agents can serve as a therapeutic option for EN in IBD patients [95].

Our patient developed EN three months after initiation of ADA therapy, which was rapidly administered in an optimized regimen and combination therapy because of poor disease control. Co-incidental is the occurrence of antibodies against ADA simultaneously with the onset of EN. Because of potential poor adherence to periodic admissions for IFX infusions and active disease, the current treatment plan was continued.

This study presents several limitations that warrant consideration. Primarily, the retrospective design inherently introduces potential biases, including incomplete or missing data in medical records. The observational nature precludes definitive causal inferences between administered treatments and observed AEs. Furthermore, unaccounted confounding variables may influence the reported associations. A significant constraint is the relatively small sample size, which substantially limits the study’s statistical power. This limitation impedes the detection of statistically significant associations between anti-TNF agent administration and outcomes. Consequently, the generalizability of our findings to broader patient populations is restricted. These methodological constraints underscore the need for larger, prospective studies to corroborate and extend our observations. Future research should aim to address these limitations to provide more robust evidence regarding the safety profile of anti-TNF agents in pediatric inflammatory bowel disease.

Despite these shortcomings, our study provides valuable observations that can enlarge our knowledge and guide future research in this field of interest.

## 4. Materials and Methods

This retrospective, single-center study recruited pediatric IBD patients diagnosed according to the ESPGHAN Porto criteria [96] from the Department of Pediatric Gastroenterology of the “Grigore Alexandrescu” Emergency Hospital for Children in Bucharest, Romania, between January 2015 and October 2024. Inclusion criteria encompassed all anti-TNF-treated patients.

This study was approved by the Ethics Committee of the “Grigore Alexandrescu” Emergency Hospital for Children in Bucharest, Romania (reference number 34/7.10.2024). The decision to initiate anti-TNF treatment was made by the treating gastroenterologist, with parental consent obtained for all patients.

Clinical and laboratory data were extracted from the hospital’s database. Key information collected included age at diagnosis and study inclusion, disease extension and behavior for UC and CD (classified according to the ESPGHAN Paris classification [97]), and time (months) from anti-TNF therapy initiation to adverse event occurrence.

Regarding the administered molecule, median dose/kg/administration and IQR were noted for IFX-treated patients. For ADA, treatment administration was divided into standard or optimized regimens (either dose escalation or weekly administration). Combination therapy was defined as the concurrent administration of an immunomodulator (IMM, specifically azathioprine) for a minimum of eight weeks preceding the initiation of biologic therapy and continuing concomitantly with anti-TNF administration. Considering the administrated molecule, for the accuracy of statistical processing, patients were divided into “IFX-only” and “IFX and/or ADA, sequential therapy” the latter comprising patients that received ADA either as first-line therapy or after IFX failure/IFX-associated AEs.

Considering the administrated molecule, for the accuracy of statistical processing, patients were divided into “IFX-only” and IFX and/or ADA, sequential therapy”.

AEs were categorized for clarity by etiology and system, according to the available literature on non-infectious complications, as hypersensitivity reactions (acute infusion reactions, anti-drug antibody formation), dermatological effects such as EN and vasculitis, neurologic effects such as acute demyelinating reactions (GBS), and infections. Minor respiratory infections that were managed locally by the general practitioner were not taken into account. Besides suggestive clinical manifestations, the AEs were confirmed on the basis of specific tests: IgM-specific antibodies for viral infections; radiology, IGRA, and polymerase chain reaction (PCR) assay from sputum for TB; and detection of *C difficile* toxins from stool with an enzyme-linked immunosorbent assay (ELISA) test. Cases of *C difficile* infection identified using glutamate dehydrogenase (GDH) and/or nucleic acid amplification tests (NAATs), without detection of toxins A and/or B, were excluded. Moreover, cases that were mild and did not require hospitalization were also excluded.

Skin biopsies were undertaken for EN and vasculitis, cerebrospinal fluid analysis, and electromyography for GBS. Anti-drug antibodies were determined by chemiluminescence immunoassay (CLIA). Hypersensitivity reactions were divided according to their severity into five severity grades, according to the National Cancer Institute [NCI] at the National Institutes of Health [NIH] in the USA. They range from mild (requiring observation only) through moderate (usually oral intervention is sufficient) and severe (vital organ involved but not life-threatening; usually requires parenteral medication) to life-threatening (multi-system involvement of vital organs, urgent and critical care required) and death [98].

### Statistics

The statistical analysis was conducted using SPSS v26, Python v3.12 (scipy.stats module), and the R platform. The choice of statistical tests was guided by the nature of the variables and the distribution of the data. For categorical variables, both the Chi-square test and Fisher’s Exact Test were employed. The Fisher’s Exact Test was preferentially used in scenarios with small sample sizes or when expected frequencies in contingency table cells fell below 5. This approach ensured robust and accurate *p*-values, particularly in cases where the assumptions of the Chi-square test might be violated. The normality of continuous variables was assessed using the Shapiro–Wilk test. Given that the data were not normally distributed, non-parametric methods were employed. The Mann–Whitney U Test was utilized to compare medians between groups. This test was selected as a non-parametric alternative to the independent samples *t*-test, as it does not assume the normality of the data. For measures of central tendency and dispersion, medians and interquartile ranges (IQR) were reported, as these are more appropriate for non-normally distributed data. A linear regression analysis was conducted to examine the relationship between age at diagnosis and time to therapy initiation. A *p*-value < 0.05 was established as the threshold for statistical significance across all analyses.

## 5. Conclusions

This study provides significant insights into the safety profile of anti-TNF agents in pediatric IBD patients. A heterogeneous spectrum of adverse reactions was observed, affecting multiple organ systems, underscoring the importance of vigilantly monitoring these patients. The occurrence of these reactions appeared to be statistically independent of concomitant medications or therapeutic strategies, suggesting an intrinsic potential of anti-TNF molecules to induce such manifestations. The potential influence of additional factors in potentiating these reactions remains a subject for further investigation, as these elements were beyond the scope of the current study.

The interpretation of AEs data associated with IFX and ADA administration necessitates a nuanced approach that considers the distribution of patients between treatment groups.

The advent of biologics has undeniably revolutionized IBD treatment, offering new therapeutic perspectives. While these agents have demonstrated efficacy in achieving prolonged steroid-free remission, improved prognosis, and enhanced quality of life, they are associated with a diverse range of potential AEs. The risk-benefit profile must be carefully considered in clinical decision-making, adhering to the fundamental principle of “primum non nocere” (first, do no harm).

In the context of rapidly evolving therapeutic options, a comprehensive understanding of the potential AEs associated with anti-TNF agents is crucial. Prospective multicenter studies are essential to validate the reported findings and explore potential associated conditions. Expanding this research to other centers will enhance our understanding of possible adverse events, ultimately leading to optimized utilization of anti-TNF molecules in pediatric IBD management.

## Figures and Tables

**Table 1:** Characteristics of the population. Data are n (%).

	CD n = 22	UC n = 18	*p*-Value
Sex [n (%)]			0.055
Male	15 (68.2%)	6 (33.3%)	
Female	7 (31.8%)	12 (66.7%)	
Age at diagnosis [n (%)]			0.110
<10 years	2 (9.1%)	6 (33.3%)	
≥10 years	20 (90.9%)	12 (66.7%)	
Age at diagnosis (years)			
Median [IQR]	15 [10.87–16.07]	14.4 [7.82–15.75]	0.145
Disease duration (years)			
Median [IQR]	1.5 [0.82–2.97]	1.85 [1.27–3.40]	0.384
UC extension			
E4		18 (100.0%)	
CD location [n (%)]			-
L1	4 (18.2%)	-	
L2	3 (13.6%)	-	
L3	15 (68.2%)	-	
L4a	7 (31.8%)	-	
CD behavior [n (%)]			-
B1	12 (54.5%)	-	
B2	8 (36.4%)	-	
B3	3 (13.6%)	-	
*p*	7 (31.8%)	-	
IMM associated [n (%)]	13 (59.1%)	12 (66.7%)	0.870
AEs [n (%)]			
Acute infusion reactions	2 (9.1%)	4 (22.2%)	0.381
Anti-drug antibodies	4 (18.2%)	8 (44.4%)	0.093
Infections	2 (9.1%)	3 (16.7%)	0.642
Dermatological reactions	1 (4.5%)	1 (5.6%)	1.0
Demyelinating reactions (GBS)	1 (4.5%)	0 (0.00%)	-
Medication administered [n (%)]			0.024
IFX	19 (86.4%) *	18 (100%)	
ADA	6 (27.3%) *	0 (0.00%)	

ADA, adalimumab; AEs, adverse events; CD, Crohn’s disease; IFX, infliximab; IMM, immunomodulator; UC, ulcerative colitis. * One patient switched from IFX to ADA because of acute infusion reaction and two patients switched from ADA to IFX because of anti-drug antibody formation; therefore, three CD patients were included in both treatment categories.

**Table 2:** Adverse events descriptive analysis.

AE	Number, % of Patients with AEs (n = 40)	Median Duration to Reaction (Months), IQR
Acute infusion reactions	6 (15)	3.5 (2.4–8.25)
Anti-drug antibodies	12 (30)	13 (9–24.5)
Infections	5 (12.5)	5 (2.5–35)
Demyelinating reactions	1 (2.5)	3
Dermatological reactions	2 (5)	61.5 (3–120)

AEs, adverse events; IQR, interquartile.

**Table 3:** Comparison in the occurrence of AEs in IFX and ADA-treated patients.

AE	IFX Group (n = 34) n, %	IFX and/or ADA Group (n = 6) n, %	*p*-Value
Acute infusion reactions			0.574
Present	6 (17.6%)	0 (0%)	
Absent	28 (82.4%)	6 (100%)	
Infections			0.128
Present	3 (8.8)	2 (33.3%)	
Absent	31 (91.2%)	4 (66.7%)	
Drug-antibodies			0.326
Present	9 (26.5)	3 (50%)	
Absent	25 (73.5%)	3 (50%)	
Demyelinating disorders			0.150
Present	0 (0)	1 (16.7)	
Absent	34 (100)	5 (83.3)	
Dermatological reactions			0.255
Present	1 (2.9)	1 (16.7)	
Absent	33 (97.1)	5 (83.3)	

ADA, adalimumab; AE, adverse event; IFX, infliximab.

**Table 4:** Adverse event occurrence according to ADA treatment regimens.

AE	Standard ADA Regimen (n = 3)	Optimized ADA Regimen (n = 3)	*p*-Value
Acute infusion reactions [n (%)]			0.273
No	3 (100%)	2 (66.7%)	
Yes	0 (0%)	1 (33.3%)	
Infections [n (%)]			0.083
No	3 (100%)	1 (33.3%)	
Yes	0 (0%)	2 (66.7%)	
Demyelinating reactions [n (%)]			0.273
No	2 (66.7%)	3 (100%)	
Yes	1 (33.3%)	0 (0%)	
Anti-drug antibodies [n (%)]			0.414
No	1 (33.3%)	2 (66.7%)	
Yes	2 (66.7%)	1 (33.3%)	
Dermatological reactions [n (%)]			0.273
No	3 (100%)	2 (66.7%)	
Yes	0 (0%)	1 (33.3%)	

ADA, adalimumab; AEs, adverse events.

## Data Availability

Data are contained within the article.
